# Effects of Yeast Cultures on Growth Performance, Fiber Digestibility, Ruminal Dissolved Gases, Antioxidant Capacity and Immune Activity of Beef Cattle

**DOI:** 10.3390/ani15101452

**Published:** 2025-05-17

**Authors:** Siyu Yi, Xu Tian, Xianwu Qin, Yan Zhang, Shuang Guan, Zhongping Chen, Daliang Cai, Duanqin Wu, Rong Wang, Zhiyuan Ma, Min Wang, Xiumin Zhang

**Affiliations:** 1 State Key Laboratory of Forage Breeding-by-Design and Utilization, National Engineering Laboratory for Pollution Control and Waste Utilization in Livestock and Poultry Production, and Hunan Provincial Key Laboratory of Animal Nutritional Physiology and Metabolic Process, Institute of Subtropical Agriculture, Chinese Academy of Sciences, Changsha 410125, China; siyuyi1996@163.com (S.Y.); rongwang@isa.ac.cn (R.W.); mwang@isa.ac.cn (M.W.); 2 Institute of Bast Fiber Crops, Chinese Academy of Agricultural Sciences, Changsha 410205, China; tx17723618850@163.com (X.T.); 15670246826@163.com (S.G.); wuduanqin@caas.cn (D.W.); 3 National Key Laboratory of Agricultural Microbiology, The Hubei Provincial Engineering Research Center of Yeast, Yichang 443000, China; qxw@angelyeast.com (X.Q.); zhangyan@angelyeast.com (Y.Z.); chenzp@angelyeast.com (Z.C.); caidl@angelyeast.com (D.C.); 4 College of Pastoral Agriculture Science and Technology, Lanzhou University, Lanzhou 730020, China

**Keywords:** yeast culture, methane, antioxidant, immune, growth performance

## Abstract

This study aimed to evaluate the effects of yeast culture (YC) supplementation on growth performance, dietary nutrient digestibility, ruminal fermentation characteristics, methane (CH_4_) synthesis potential, ruminal bacterial composition, antioxidant and immune capacities in beef cattle. Thirty-six finishing Simmental beef cattle were employed for this experiment. The experiment included three dietary treatments: the basal diet (CON), the basal diet supplemented with Diamon V XP (XP; 50 g/day per cattle), and the basal diet supplemented with Keliben (KLB; 50 g/day per cattle). Various metabolites, such as acetophenone (12.7%), ascorbic acid (10.3%), citric acid (7.25%), D-(+)-proline (6.42%), succinic acid (5.70%), betaine (5.65%) and DL-malic acid (2.62%) were abundant in XP; and ascorbic acid (14.0%), oleamide (9.23%), citric acid (6.03%), betaine (5.88%), succinic acid (4.42%), indole-3-acrylic acid (2.85%) and DL-malic acid (1.73%) were abundant in KLB. Supplementing YC to the cattle increased the apparent total-tract digestibility of DM, OM, NDF and ADF, and tended to increase average daily gain. The supplementation of YC to the cattle had no effect on the rumen fermentation pathway as reflected by the unaltered molar percentage of acetate or propionate. The supplementation of XP decreased the concentration of rumen dissolved CH_4_, although no effect on the concentration of dissolved hydrogen was observed. The supplementation of KLB to the cattle increased *Paraprevotella* relative abundance, while the supplementation of XP decreased Euryarchaeota relative abundance in the rumen. Supplementing XP to the cattle increased serum GSH-Px, catalase and T-AOC concentrations, and was accompanied by decreased MAD concentration, indicating improved antioxidant capacity. Supplementing XP to the cattle increased the concentrations of serum IgA, IgG, IL-2, IL-10, IFN-γ and C4, compared with the CON group, and the KLB group had higher concentrations of serum IgA, IgG, IgM, IL-10, IFN-γ, C3 and C4 compared with the CON and XP groups, which revealed that both XP and KLB can improve the immune function, and that KLB showed a stronger effect. Overall, the supplementation of YC is beneficial to the nutrient digestibility, growth performance and health of beef cattle. Furthermore, XP was more effective than KLB in improving antioxidant capacity and reducing CH_4_ production, while KLB was more effective in improving the immune capacity of beef cattle than XP.

## Simple Summary

Yeast culture (YC) has been widely used as an additive in ruminants, and has various functions of improving growth performance, nutrient digestibility and antioxidant and immune capacities. However, the mechanism of metabolic components in YC and their inhibitory potential on methane (CH_4_) emissions from ruminants are still unclear. This study explored supplementation with Diamon V (XP) and Keliben (KLB) to the diet of finishing Simmental beef cattle and found that XP was more effective in improving antioxidant capacity and reducing CH_4_ production, and that KLB was more effective in improving the immune capacity. Thus, YC is an effective additive for improving production performance and health, with potential for the suppression of CH_4_ production in beef cattle.

## 1. Introduction

Yeast culture (YC) is a natural fermentation product produced by *Saccharomyces cerevisiae* in anaerobic environments, and it has been widely used as an additive in ruminants [1,2,3]. It has been found that YC plays a beneficial role in the growth performance of beef cattle, which may be attributed to the improvement of dietary neutral detergent fiber (NDF) and crude protein (CP) digestibility [4]. Moreover, Chen et al. [5] found that YC can improve the serum total antioxidant capacity (T-AOC) and catalase and glutathione peroxidase (GSH-Px) concentrations of lambs. Chen et al. [5] and Li et al. [6] found that the supplementation of YC to ruminants increased the levels of immunoglobulin A (IgA), immunoglobulin G (IgG), immunoglobulin M (IgM) and interferon-γ (IFN-γ) in blood, which means that YC can enhance the antioxidant capacity and immunity of ruminants. However, Zhang et al. [7] found that the supplementation of YC had no effects on the immunity of cattle, which may be caused by the differences in effective components of different YC. Yeast culture includes the cells and metabolites of *Saccharomyces cerevisiae* and culture medium components, including organic acids, mannan, β-glucan, active peptides, nucleotides, vitamins, oligosaccharides, etc. [1], which may contribute to the various biological functions of YC. However, it is not clear how the specific metabolites in YC affect the growth performance and health of animals.

The enteric methane (CH_4_) of ruminants is an important anthropogenic greenhouse gas emission source globally, which is accompanied by a large amount of energy loss of ruminants, which reduces the breeding efficiency [8,9]. Hydrogen (H_2_) is produced during carbohydrate fermentation and is mainly utilized by methanogens in the dissolved form to produce CH_4_ in the rumen [10]. The remaining H_2_ can be reduced to other reduction products, such as microbial protein (MCP) and propionate, or emitted in gaseous form of [10,11]. The amount of H_2_ production is usually determined by the volatile fatty acids (VFA) production pattern in the rumen, and the formation of acetate and propionate leads to its production and consumption, respectively [9]. Although previous studies have found that [12,13] active dried *Saccharomyces cerevisiae* did not reduce CH_4_ emissions in ruminants, the potential and mechanism of CH_4_ emission reduction of YC need to be further explored. The supplementation of YC usually unalters the fermentation characteristics of ruminal VFA in ruminants, such as the molar percentages of propionate, acetate and butyrate [2,14], which means that YC supplementation may not reduce enteric CH_4_ production by reducing the H_2_ production. Methanogenic archaea dominate the synthesis of CH_4_ in the rumen, and inhibiting its activity is also beneficial to CH_4_ emission reduction [15]. Therefore, it is necessary to further explore the CH_4_ inhibition effects of YC by combining the VFA profile and the relative abundance of methanogenic archaea in the rumen.

Herein, our purpose is to explore the effects of YC on growth performance and health of beef cattle, and its potential to inhibit methanogenesis in the rumen. Thus, we analyzed the nutrient and metabolite components of two different YC, Diamond V XP (XP) and Keliben (KLB), and explored the effects of YC on the growth performance, dietary nutrients digestibility, ruminal fermentation characteristics and dissolved gases, ruminal bacterial composition and antioxidant and immune capacities of beef cattle.

## 2. Materials and Methods

### 2.1. Sources of YC

The samples of XP and KLB were purchased from Diamond V Bio-fermentation Technology Co., Ltd., Shenzhen, China and Angel yeast Co., Ltd., Yichang, China, respectively, and were stored in sealed bags at room temperature.

### 2.2. Animals, Experimental Design and Feeding

A total of 36 finishing male Simmental beef cattle with similar age (18.3 ± 0.70 months) and weight (460 ± 17.4 kg) were randomly divided into 3 groups: CON (the basal diet; Table 1), XP (the basal diet supplemented with 50 g/day per cattle of XP) and KLB (the basal diet supplemented with 50 g/day per cattle of KLB), with 12 cattle in each group. The experiment was conducted on a beef cattle farm of Xindalinmu Science and Technology Development Co., Ltd., Yuanjiang, Yiyang, Hunan, China (29°07′ N, 112°34′ E). Cattle were kept in separate pens, fed with total mixed rations (TMR; mixed by a horizontal mixer) at 07:00 and 16:00 every day with an independent feeder, and were free to drink water. Yeast culture was evenly top-dressed on the TMR diet of each cattle. The experiment lasted for 70 days (including 10 days of a dietary adaptation period).

### 2.3. Apparent Total-Tract Digestibility, Nutrient Components and Growth Performance

During the 11–70th days of the experimental period, the dry matter intake (DMI) was calculated by recording the feed intake and refusals daily. During the 60–64th days of the experimental period, about 200 g fecal samples were collected from the rectum at 08:00 and 17:00 and divided into two subsamples on average. One subsample was added with 10 mL 10% (*v*/*v*) dilute sulfuric acid for nitrogen fixation to determine CP, and the other subsample was used to determine other nutrient components, and then immediately stored at −20 °C. After that, the fecal samples were dried and crushed at 65 °C, and the samples of the same cattle for 5 days were mixed in equal proportions with the DMI and then stored in a sealed bag. Feed samples were randomly collected every 7 days, mixed and dried at 65 °C, and kept in sealed bags for determination. On the 34th and 70th days of the experimental period, all cattle were weighed before the morning feeding, and the average daily gain (ADG) was calculated.

The dry matter (DM; method 934.01), organic matter (OM; method 942.05) and CP (method 990.03) contents were determined according to the AOAC [17]. The gross energy (GE) was measured by an isothermal automatic calorimeter (5E-AC8018; Changsha kaide measurement and control instruments Co., Ltd., Changsha, China). The contents of NDF and acid detergent fiber (ADF) were determined according to the method previously described by Van Soest et al. [18], and the heat-stable amylase and sodium sulfite were added when the NDF was determined. The starch content was determined following the method previously described by Kartchner et al. [19]. The acid-insoluble ash content of feed and feces samples was determined in accordance with the GB/T 23,742 method [20], and the apparent total-tract digestibility of nutrients was calculated according to the method described by Van Keulen et al. [21] with the acid-insoluble ash contents.

Mannan content in YC was analyzed according to T/QBAA 001 method [22] by high-performance liquid chromatography (HPLC; LC1290; Agilent Inc., Santa Clara, CA, USA). The contents of free amino acids, acid-soluble protein and total organic acids in YC were analyzed according to GB/T 18,246 [23], NY/T 3801 [24] and GB/T 12,456 [25] methods. The metabolites content of YC was determined by Nuohezhiyuan Technology Co., Ltd., Beijing, China. The content of metabolites was analyzed by a liquid chromatography–mass spectrometer (LC-MS; Agilent Inc., Santa Clara, CA, USA) [26,27]. The original data of metabolite determination are preprocessed by Compound Discoverer 3.1 data processing software. Then, the metabolites were identified by comparing the high-resolution secondary spectrogram databases of mzCloud and mzVault with the primary database of MassList. Metabolites with a coefficient of variance less than 30% in the samples were retained as the final result [28]. The chromatographic peaks detected in the samples are integrated, in which the peak area of each characteristic peak represents the relative quantitative value of a metabolite, and the peak area quantitative result of each characteristic peak is standardized by using the total peak area. The analysis of the nutrients and metabolites components was performed in triplicate and sextuplicate, respectively.

### 2.4. Collection and Determination of Ruminal Contents

During the 67–70th days of the experimental period, ruminal contents were taken from the middle of the rumen at 0 and 2.5 h after feeding in the morning through a rumen cannula. About 100 mL of ruminal contents collected at the beginning were discarded to avoid saliva pollution. The pH value of ruminal contents was measured immediately by a pH meter (Starter 300; Ohaus Instruments Co., Ltd., Shanghai, China). The dissolved hydrogen (dH_2_), dissolved CH_4_ (dCH_4_) and VFA concentrations were analyzed as described by Wang et al. [29], by a gas chromatograph (7890A; Agilent Inc., Santa Clara, CA, USA). Briefly, 35 mL of ruminal contents were transferred to a 50 mL-plastic syringe, and the dissolved gases in ruminal contents were injected into another connected 20 mL-syringe with 10 mL of nitrogen by a shaker (Huichen Biotech Co., Ltd., Wuhan, China), and the gases were collected into a sealed bottle for determination. About 2 mL of ruminal contents were centrifuged at 12,000× *g* for 10 min at 4 °C, and the supernatants (1.5 mL) were acidified with 0.15 mL of 25% (*w*/*v*) metaphosphoric acid and stored at −20 °C for the determination of VFA. About 5 mL of ruminal contents were immediately frozen in liquid nitrogen and then stored at −20 °C for MCP content analysis according to the method described by Zinn et al. [30]. In addition, about 2 mL of ruminal contents were immediately frozen in liquid nitrogen and then stored at −80 C for subsequent DNA extraction.

### 2.5. Collection and Determination of Blood Samples

On the 65–66th days of the experimental period, blood samples were collected through the jugular vein at 2 h before feeding in the morning using a 5 mL vacuum blood collection tube. The collected blood was immediately transferred to an incubator for incubation for 30 min, then centrifuged at 3000× *g* for 15 min at room temperature to separate the serum samples and stored at −20 °C.

Serum metabolites were determined by Roche automatic biochemical analyzer (cobas c311; Roche Inc., Basel, Switzerland). The serum antioxidant index and immune index were determined by unitized biochemical and ELISA kits (Nanjing jiancheng bioengineering institute, Nanjing, China), respectively.

### 2.6. Analysis of Ruminal Microbiota Composition

Samples of ruminal contents collected at 0 h and 2.5 h after feeding were mixed, and the total microbial DNA was extracted according to Ma et al. [31]. The 16S rDNA sequencing was carried out by Biozeron Biotechnology Co., Ltd., Shanghai, China, on the MiSeq platform (Illumina Inc., San Diego, CA, USA), and the PCR amplification process was as described by Zakrzewski et al. [32]. The universal primer of 341F: (5′-CCTAYGGGRBGCASCAG-3′) and 806R: (5′-GGACTACNNGGGTATCTAAT-3′) was used to amplify the V3-V4 region of bacteria. The operational taxonomic units were clustered with a 98.7% similarity cutoff by UPARSE software (version 7.1; http://drive5.com/uparse/ (accessed on 6 July 2024)), and UCHIME was used to identify and remove the chimeric sequences. The phylogenetic affiliation of each 16S rRNA gene sequence was analyzed using RDP Classifier (http://rdp.cme.msu.edu/ (accessed on 6 July 2024)) against the 16S rRNA database of silva (SSU132) with a 70% confidence threshold [33]. The analysis of microbiota composition was performed in twelve duplicates.

### 2.7. Statistical Analysis

The data were analyzed using a linear mixed model by SPSS 26.0 software (SPSS Inc., Chicago, IL, USA), with the groups (CON, XP and KLB) as a fixed effect, and the animal as a random effect. When the sampling time is included, the groups and interaction between groups and sampling times were used as the fixed effects, the animal as a random effect, and the sampling time as a repeated measurement. The data of bacterial compositions were analyzed by nonparametric test models, and the differences were judged by the Kruskal–Wallis test. The statistical significance was set at *p* < 0.05, and *p* < 0.10 is considered to have a difference trend.

## 3. Results

### 3.1. Nutrient Components and Metabolites Components of YC

The nutrient components of XP and KLB are shown in Table 2. The content of mannan was 90.0 and 140 g/kg of DM, respectively. The metabolites components (the top 10) of XP and KLB are presented in Table 3. Many substances with antioxidant activity and immunity enhancement were found to be abundant in YC, such as acetophenone (12.7%), ascorbic acid (10.3%), citric acid (7.25%), D-(+)-proline (6.42%), succinic acid (5.70%), betaine (5.65%) and DL-malic acid (2.62%) in XP; and ascorbic acid (14.0%), oleamide (9.23%), citric acid (6.03%), betaine (5.88%), succinic acid (4.42%), indole-3-acrylic acid (2.85%) and DL-malic acid (1.73%) in KLB.

### 3.2. Growth Performance and Apparent Total-Tract Digestibility

The supplementation of XP and KLB increased (*p* < 0.01) the apparent total-tract digestibility of DM, OM, NDF, ADF and GE of beef cattle, and tended to increase (*p* = 0.06) starch digestibility of beef cattle. Moreover, the supplementation of XP increased (*p* < 0.01) CP digestibility. Consequently, XP and KLB supplementation showed a trend of increased (*p* = 0.06) ADG (Table 4).

### 3.3. Dissolved Gases and Ruminal Fermentation Characteristics

Supplementing YC had no effect (*p* > 0.10; Table 5) on rumen dH_2_ concentration, whereas XP decreased (*p* = 0.04) the dCH_4_ concentration in the rumen. The cattle fed with XP showed a trend (*p* = 0.08) for increasing MCP concentration in the rumen. Both XP and KLB supplementation had no effects (*p* > 0.10) on ruminal pH, acetate to propionate ratio and the molar percentages of acetate, propionate, butyrate and valerate. Interaction (*p* = 0.02) between groups and sampling times was observed for total VFA concentration. The cattle fed with KLB tended to decrease (*p* = 0.06) the total VFA concentration compared with the CON group before the morning feeding, while XP supplementation decreased (*p* < 0.01) the total VFA concentration compared with the CON and KLB groups at 2.5 h after the morning feeding. Interaction (*p* < 0.01) between groups and sampling times was observed for isobutyrate concentration. The supplementation of XP and KLB increased (*p* < 0.01) isobutyrate molar percentages before the morning feeding, but had no effect (*p* > 0.10) at 2.5 h after morning feeding between the groups. Interaction (*p* < 0.01) between groups and sampling times was observed for isobutyrate concentration. The supplementation of XP and KLB increased (*p* < 0.01) isovalerate molar percentages before the morning feeding, but had no effect (*p* > 0.10) at 2.5 h after morning feeding between the groups.

### 3.4. Bacterial Composition in the Rumen

**Figure 1:**
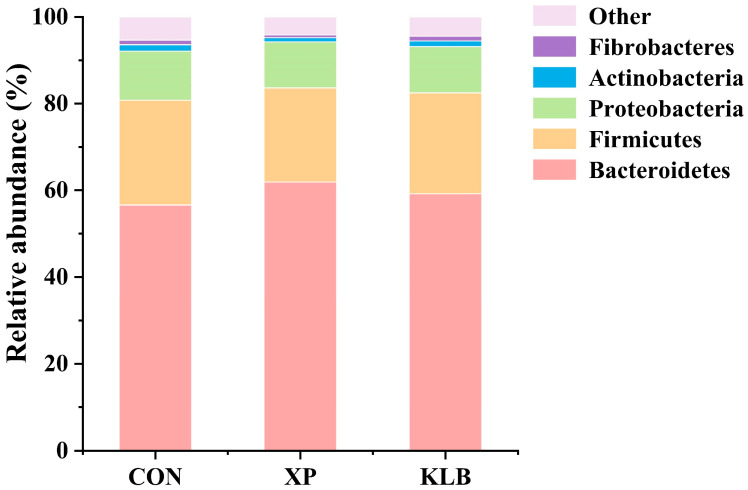
Effect of yeast cultures supplementation on the relative abundance of ruminal bacteria at the phyla level of finishing beef cattle.

The composition of bacteria in the rumen at phylum level (>1.00%) is shown in Figure 1. No differences between the groups were observed in the relative abundances (CON vs. XP vs. KLB) of Bacteroidetes (56.6% vs. 61.9% vs. and 59.2%), Firmicutes (24.2% vs. 21.7% vs. 23.3%), Proteobacteria (11.3% vs. 10.6% vs. 10.7%), Actinobacteria (1.49% vs. 0.95% vs. 1.30%) and Fibrobacteres (1.05% vs. 0.62% vs. 1.06%) in the rumen (*p* > 0.10).

**Figure 2:**
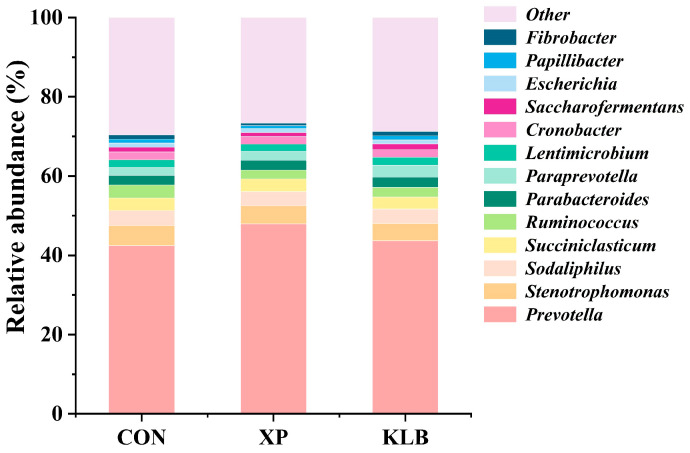
Effect of yeast cultures supplementation on the relative abundance of ruminal bacteria at the genera level of finishing beef cattle.

The composition of bacteria in the rumen at the genus level (>1.00%) is presented in Figure 2. The supplementation of KLB increased (*p* = 0.04) the relative abundance of *Paraprevotella* (1.87% vs. 2.21% vs. 2.96%; CON vs. XP vs. KLB). However, no differences (*p* > 0.10) between the groups were observed in *Prevotella* (42.4% vs. 48.0% vs. 43.7%), *Stenotrophomonas* (5.08% vs. 4.55% vs. 4.40%), *Sodaliphilus* (3.81% vs. 3.57% vs. 3.61%) and *Succiniclasticum* (3.14% vs. 3.15% vs. 3.02%).

**Figure 3:**
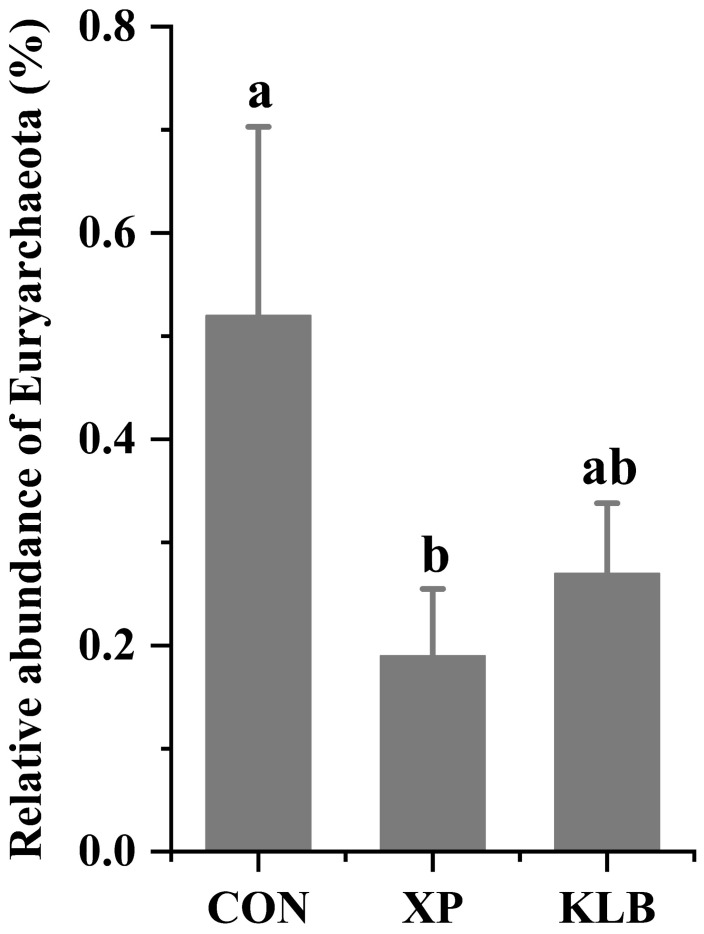
Effect of yeast culture supplementation on the relative abundance of ruminal Euryarchaeota in finishing beef cattle. Different letters indicate significant differences (*p* < 0.05).

The relative abundance of Euryarchaeota in the rumen is shown in Figure 3. The cattle fed with XP had a lower relative abundance of (*p* = 0.03) Euryarchaeota compared with the cattle in the CON group.

### 3.5. Serum Biochemical Indices

In terms of serum carbohydrate metabolism capacity, supplementing XP and KLB increased (*p* ≤ 0.01; Table 6) the concentrations of glucose, α-amylase and pancreatic amylase, but decreased (*p* < 0.01) lactic acid concentration. For serum nitrogen metabolism capacity, the supplementation of XP and KLB decreased (*p* = 0.02) uric acid concentration, whereas it increased (*p* < 0.01) creatinine concentration. Furthermore, the KLB group had higher (*p* < 0.01) serum concentrations of BUN and alanine aminotransferase (ALT) than the CON and XP groups, and had a higher (*p* < 0.01) albumin concentration in serum than the CON group. In terms of serum lipid metabolism capacity, XP supplementation increased (*p* = 0.02) lipase concentration, whereas the supplementation of XP and KLB had no effects (*p* ≥ 0.10) on total cholesterol, triglyceride, low-density lipoprotein cholesterol (LDL-C) and high-density lipoprotein cholesterol (HDL-C) concentrations.

### 3.6. Serum Antioxidant and Immune Indexes

The effects of XP and KLB supplementation on the serum antioxidant and immune capacities of beef cattle are presented in Table 7. With regard to serum antioxidant indexes, the XP group had higher (*p* < 0.05) GSH-Px, catalase and T-AOC concentrations compared with the CON and KLB groups. The cattle fed with XP had a lower (*p* = 0.04) malondialdehyde (MAD) concentration compared with the CON group. However, there was no difference (*p* > 0.10) in superoxide dismutase (SOD) between the CON, XP and KLB groups. In terms of immunoglobulins, the XP group had higher (*p* < 0.01) IgA and IgG concentrations than the CON group, and the cattle fed with KLB had higher (*p* < 0.01) IgA, IgG and IgM concentrations than the CON and XP groups. For immune factors, supplementation with XP and KLB increased (*p* < 0.01) IL-2 concentration, and the cattle fed with KLB had higher (*p* < 0.01) interleukin-10 (IL-10), IFN-γ, complement C3 (C3) and complement C4 (C4) concentrations in serum compared with the CON and XP groups. Furthermore, the cattle fed with XP had higher (*p* < 0.01) IL-10, IFN-γ and C4 concentrations in serum than the CON group.

## 4. Discussion

Yeast culture has been reported to improve the growth performance, nutrient digestibility, antioxidant capacity and immune function of beef cattle [4,6]. These functions of YC may be related to its rich active substances, such as organic acids, mannan, β-glucan, active peptides, nucleotides and some undiscovered growth factors [1]. In the present study, various metabolites, such as betaine, ascorbic acid, citric acid, succinic acid, malic acid, acetophenone, oleamide and indole-3-acrylic acid were found to be enriched in YC, which may also improve the health and then promote the growth performance of livestock.

Yeast culture can improve the nutrients digestibility, which is related to the improvement of the activities of beneficial microorganisms and digestive enzymes [34,35]. The supplementation of XP and KLB into the diet of beef cattle resulted in a significant increase in the digestibility of NDF and ADF. Consistent with the present study, Wang et al. [2] supplemented YC (10–40 g/d/head) in the diet of fattening sheep and improved the digestibility of NDF and ADF. In addition, supplementation of XP increased the CP digestibility of beef cattle, while KLB supplementation did not, which may be related to the change in rumen microbial community. *Prevotella* is usually the dominant bacteria in the rumen and plays an important role in the degradation of protein [36]. In the present study, the supplementation of XP increased the relative abundance (48.0%) of *Prevotella* in the rumen compared with the CON (42.4%) and KLB (43.7%) groups, which helps uphold the improved CP digestibility. Thus, supplementing YC can promote the digestion of dietary nutrients, which may further improve the growth performance of beef cattle, as demonstrated by the increased ADG.

The total rumen VFA concentration is closely related to the digestion of dietary nutrients. However, the supplementation of XP and KLB did not increase the total VFA concentration as expected, XP even reduced the VFA concentration. One possible reason is that YC promoted the absorption of VFA by rumen epithelium [2,37]. We found that the supplementation of XP and KLB unaltered the molar percentages of propionate, acetate and butyrate and the acetate to propionate ratio. Our results are consistent with Wang et al. [2], who reported that the supplementation of YC (10–40 g/d/head) to sheep did not change the rumen fermentation pathway. However, supplementing XP and KLB to beef cattle increased isobutyrate molar percentages before the morning feeding. Ruminal isobutyrate is mainly derived from the degradation of dietary amino acids, i.e., valine, isoleucine, leucine and proline [38], and the higher contents of valine and proline in XP and KLB could contribute to the increased production of isobutyrate. Furthermore, the cattle fed with XP tended to have a higher MCP concentration compared with the CON group, which is consistent with the fact that XP supplementation enhanced amino acids availability for microbes, and the increased CP digestibility due to XP supplementation can also contribute to the enhanced MCP synthesis.

Ruminal H_2_ is produced during the carbohydrates fermentation process and exists as a gaseous or dissolved form, and only the dH_2_ can be utilized by the microbes. The dH_2_ concentration is determined by the balance between H_2_ production pathways (e.g., acetate production from carbohydrate fermentation) and consumption pathways (e.g., CH_4_, propionate and MCP formation) in the rumen, and methanogenesis makes the greatest contribution to the H_2_ consumption [9,10,11]. Interestingly, we found that the concentration of dCH_4_ in the rumen decreased after XP supplementation, which seems to indicate that methanogenesis was inhibited. Moreover, Euryarchaeota, as the main methanogenic archaea in the rumen [39], was found to be significantly decreased after XP supplementation, which is consistent with the decreased dCH_4_ concentration. However, the dH_2_ concentration was not increased after methanogenesis inhibition. These results may indicate that the supplementation of XP inhibited methanogenesis, which may lead to more H_2_ being redirected to other reduction products, such as the MCP synthesis, which was likely demonstrated by the increased MCP concentration. Therefore, we speculated that XP supplementation inhibited the activity of methanogens, which may lead to more H_2_ being utilized to form other reduction products (e.g., MCP), rather than CH_4_.

Serum metabolites are an important indicator of nutrient digestion and body metabolism [40]. In the present study, the supplementation of XP and KLB increased serum glucose concentration, which was consistent with the higher digestibility of fiber and starch. In addition, XP and KLB supplementation had no effects on total protein, total cholesterol, blood ammonia, total cholesterol, triglyceride, LDL-C and HDL-C concentrations in serum compared with the CON group, which means that YC had no negative impacts on body metabolism. Although KLB supplementation increased the serum contents of albumin, creatinine and ALT, it was still within the normal physiological range [41].

The animal organism continues to carry out oxidation reactions to meet the needs of normal metabolism of the substances of life. However, when the level of reactive oxygen free radicals exceeds the upper limit of degradation ability (often caused by feed, environment and social factors), it can lead to oxidative stress, endangering animal health and having a negative impact on production efficiency [42]. Serum SOD, GSH-Px, catalase and T-AOC are important indexes to measure the antioxidant performance of animals, while serum MAD concentration can reflect the degree of oxidative damage to the animals [42]. In the present study, the supplementation of XP increased the concentration of GSH-Px, catalase and T-AOC, and decreased the concentration of MAD, indicating that XP supplementation can enhance the antioxidant capacity of beef cattle. Acetophenone, which can improve antioxidant activity, was found to be abundant in XP rather than KLB [43], which may contribute to the improvement of antioxidant capacity in beef cattle.

Serum IgA, IgG and IgM play a pivotal role in both the acquired response and the innate immune systems [44], and IL-2, IL-10, IFN-γ, C3 and C4 play an important role in maintaining physiological balance and innate immunity, regulating inflammation, preventing immune hyperresponsiveness, promoting T cell proliferation and differentiation, and resisting virus invasion [45,46,47,48]. In the present study, we found that supplementation with XP increased the concentrations of IgA, IgG, IL-2, IL-10, IFN-γ and C4 in the serum compared with the CON group, while KLB supplementation increased the concentrations of IgA, IgG, IgM, IL-10, IFN-γ, C3 and C4 compared with the CON and XP groups, and increased IL-2 concentration compared with the CON group. These results indicated that YC enhances the immune response of cattle, and KLB is more effective than XP. The metabolites, such as betaine, ascorbic acid, citric acid, succinic acid and malic acid, were abundant in XP and KLB, and have the functions of improving immunity [49,50,51,52,53], which may be the contributing factor to the enhanced immunity observed in cattle. Mannan, oleamide and indole-3-acrylic acid, abundant in KLB rather than XP, have been found to enhance the immunity in animals [54,55,56], which may promote KLB to be more effective than XP in improving immunity. In addition, *Paraprevotella* in the rumen has been found to be related to immune function [57,58], thus the greater relative abundance of ruminal *Paraprevotella* by KLB supplementation can help explain the improved immunity of beef cattle.

## 5. Conclusions

Supplementation with YC enhances the nutrient digestion (e.g., fiber), antioxidant capacity and immunity of beef cattle. Compared with KLB, XP improves the CP digestibility and antioxidant capacity and demonstrates the potential to reduce CH_4_ production. However, KLB exhibits stronger immune-boosting effects than XP. In summary, YC is an effective additive for improving the production performance and health of beef cattle, with the potential for rumen methanogenesis suppression, which could be considered in the diets of beef cattle to enhance productivity and reduce environmental impact.

## Figures and Tables

**Table 1:** Composition of ingredients and nutrients of the basal diet, as dry matter basis.

Ingredients ^1^	Content, %	Nutrients ^2^	Content, g/kg
Corn stover silage	45.0	OM	909
Corn	23.1	CP	113
Soybean meal	5.50	NDF	476
Wheat bran	4.40	ADF	350
Cron gluten	4.40	Starch	165
DDGS	8.25	GE, Mcal/kg	4.25
Cotton meal	2.20	NEm, Mcal/kg	2.47
Molasses	1.10	NEg, Mcal/kg	1.49
Rice bran meal	2.75		
NaHCO_3_	0.55		
Premix	2.75		

^1^ Premix contains 250,000 IU of vitamin A, 70,000 IU of vitamin D3, vitamin E 1000 IU, 0.8 g of copper, 1.5 g of zinc, 1.3 g of manganese, 27 mg of iodine, 11.5 mg of selenium, 23 mg of cobalt, 17% of calcium and 3.5% of phosphorus per kg premix. ^2^ OM: organic matter; CP: crude protein; NDF: neutral detergent fiber; ADF: acid detergent fiber; GE: gross energy; NEm: net energy for maintenance; NEg: net energy for gain. The values of NEm and Neg were calculated according to NY/T 815 [16].

**Table 2:** Nutrient components of yeast cultures, as dry matter basis.

Items	XP	KLB
OM, g/kg	902	895
CP, g/kg	171	167
Mannan, g/kg	90.0	140
Free amino acid, g/kg	7.40	23.7
Total organic acid, g/kg	88.6	90.9
Acid-soluble protein, g/kg	56.0	55.4
GE, MJ/kg	18.4	17.5

OM: organic matter; CP: crude protein; GE: gross energy.

**Table 3:** The metabolites components (the top 10) of yeast cultures detected by positive and negative ions mode.

Metabolites of XP	Content, %	Metabolites of KLB	Content, %
Positive ion			
Acetophenone	12.7	Oleamide	9.23
D-(+)-Proline	6.42	Betaine	5.88
DL-3-Hydroxynorvaline	6.38	L-Pyroglutamic acid	5.43
Saxitoxin	6.05	9-Oxo-10(E),12(E)-octadecadienoic acid	5.21
Betaine	5.65	Indole-3-acrylic acid	2.85
LPC 16:0	3.46	Valine	2.26
N6-Me-dA	3.14	DL-3-Hydroxynorvaline	2.15
Valine	3.12	D-(+)-Proline	2.12
L-Pyroglutamic acid	2.50	L-Tyrosine	1.98
Tyramine	2.41	Deoxycorticosterone 21-glucoside	1.97
Negative ion			
Ascorbic acid	10.3	Ascorbic acid	14.0
(+/−)12(13)-DiHOME	10.1	(+/−)12(13)-DiHOME	13.8
Citric acid	7.25	Citric acid	6.03
Succinic acid	5.70	2-Isopropylmalic acid	4.44
5-Methoxypsoralen	5.65	Succinic acid	4.42
(+/−)9-HpODE	4.58	4-Oxoproline	3.83
4-Oxoproline	3.69	(+/−)9-HpODE	3.68
DL-Malic acid	2.62	2-Hydroxyvaleric acid	1.76
2-Isopropylmalic acid	2.62	DL-Malic acid	1.73
cis-Aconitic acid	2.43	2-Hydroxycaproic acid	1.56

**Table 4:** Effects of yeast cultures supplementation on performance and apparent total-tract digestibility of beef cattle.

Items	Groups			SEM	*p*-Value
	CON	XP	KLB		
Performance					
IBW, kg	465	454	460	17.4	0.81
FBW, kg	488	480	493	16.2	0.72
ADG, kg/d	0.63	0.72	0.92	0.11	0.06
DMI, kg/d	10.8	10.7	10.8	0.08	0.30
Apparent total-tract digestibility, g/kg					
DM	651 ^b^	710 ^a^	686 ^a^	6.00	<0.01
OM	685 ^b^	743 ^a^	723 ^a^	6.19	<0.01
CP	593 ^b^	669 ^a^	620 ^b^	8.81	<0.01
NDF	615 ^b^	689 ^a^	691 ^a^	9.52	<0.01
ADF	397 ^b^	503 ^a^	535 ^a^	17.2	<0.01
Starch	960	968	970	1.85	0.06
GE, KJ/MJ	657 ^b^	718 ^a^	694 ^a^	6.52	<0.01

IBW: initial body weight; FBW: final body weight; ADG: average daily gain; DMI: dry matter intake; DM: dry matter; OM: organic matter; CP: crude protein; NDF: neutral detergent fiber; ADF: acid detergent fiber; GE: gross energy. Different letters indicate significant differences between the groups (*p* < 0.05).

**Table 5:** Effects of yeast cultures supplementation on dissolved gases and fermentation end-products in the rumen of beef cattle.

Items	Group			Time, h		SEM	*p*-Value		
	CON	XP	KLB	0	2.5		Group	Time	Group × Time
dH_2_, μM	0.14	0.18	0.16	0.14	0.18	0.01	0.31	0.03	0.63
dCH_4_, mM	0.26 ^a^	0.21 ^b^	0.24 ^ab^	0.22	0.25	0.01	0.04	0.07	0.11
MCP, g/L	1.49	2.04	1.67	1.94	1.53	0.11	0.08	0.06	0.53
pH	7.16	7.16	7.25	7.51	6.86	0.05	0.23	<0.01	0.54
Total VFA, mM	80.4 ^a^	69.8 ^b^	73.3 ^ab^	57.3	91.7	2.57	0.03	<0.01	0.02
Molar percentage of individual VFA, mol/100 mol									
Acetate	65.7	66.1	65.8	68.2	63.6	0.58	0.36	<0.01	0.49
Propionate	18.9	19.0	18.7	17.4	20.4	0.29	0.95	<0.01	0.27
Butyrate	12.0	11.1	11.6	0.34	10.2	0.22	0.10	<0.01	0.26
Isobutyrate	0.94 ^b^	1.15 ^a^	1.11 ^a^	1.41	0.73	0.05	0.01	<0.01	<0.01
Valerate	1.15	1.08	1.24	0.94	1.38	0.04	0.21	<0.01	0.34
Isovalerate	1.31	1.57	1.52	1.89	1.05	0.06	0.05	<0.01	<0.01
Acetate to propionate ratio	3.53	3.55	3.61	3.97	3.15	0.12	0.86	<0.01	0.23

dH_2_: dissolved hydrogen; dCH_4_: dissolved methane; MCP: microbial protein; VFA: volatile fatty acids. Different letters indicate significant differences between the groups (*p* < 0.05).

**Table 6:** Effects of yeast cultures supplementation on serum metabolites of finishing beef cattle.

Items	Groups			SEM	*p*-Value
	CON	XP	KLB		
Carbohydrate metabolism					
Glucose, mM	3.26 ^b^	4.57 ^a^	4.81 ^a^	0.17	<0.01
Lactic acid, mM	4.80 ^a^	2.34 ^b^	2.49 ^b^	0.30	<0.01
Lactate dehydrogenase, U/L	1105	1180	1181	33.4	0.57
α-amylase, U/L	27.7 ^b^	40.9 ^a^	45.6 ^a^	2.61	<0.01
Pancreatic amylase, U/L	27.3 ^b^	39.2 ^a^	43.6 ^a^	2.47	0.01
Nitrogen metabolism					
Total protein, g/L	74.3	75.7	79.2	1.20	0.22
Albumin, g/L	34.7 ^b^	38.2 ^ab^	39.8 ^a^	0.69	<0.01
blood ammonia, μM	248	195	226	12.9	0.24
Uric acid, mg/dL	0.60 ^a^	0.44 ^b^	0.43 ^b^	0.03	0.02
BUN, mM	4.33 ^b^	4.94 ^b^	5.92 ^a^	0.18	<0.01
Creatinine, μM	101 ^b^	140 ^a^	130 ^a^	3.85	<0.01
AST, U/L	82.3	70.0	79.7	3.37	0.21
ALT, U/L	24.5 ^b^	25.2 ^b^	30.3 ^a^	0.84	<0.01
Lipid metabolism					
Total cholesterol, mM	2.60	2.58	2.72	0.16	0.76
Triglyceride, mM	0.28	0.24	0.27	0.01	0.10
LDL-C, mM	0.88	0.80	0.92	0.05	0.59
HDL-C, mM	2.08	2.11	2.23	0.08	0.65
Lipase, U/L	16.9 ^b^	21.1 ^ab^	22.7 ^a^	0.90	0.02

BUN: blood urea nitrogen; AST: aspartate aminotransferase; ALT: alanine aminotransferase; LDL-C: low-density lipoprotein cholesterol; HDL-C: high-density lipoprotein cholesterol. Different letters indicate significant differences between the groups (*p* < 0.05).

**Table 7:** Effects of yeast cultures supplementation on serum antioxidant and immune indexes of finishing beef cattle.

Items	Groups			SEM	*p*-Value
	CON	XP	KLB		
Antioxidants					
SOD, U/mL	315	314	307	5.96	0.85
GSH-Px, U/mL	30.0 ^b^	38.0 ^a^	28.9 ^b^	2.69	<0.01
Catalase, U/mL	0.45 ^b^	0.68 ^a^	0.47 ^b^	0.04	<0.01
T-AOC, U/mL	1.18 ^b^	1.51 ^a^	1.21 ^b^	0.07	0.04
MAD, nmol/mL	5.12 ^a^	3.27 ^b^	3.88 ^ab^	0.32	0.04
Immunoglobulins					
IgA, mg/mL	1.62 ^c^	1.91 ^b^	2.52 ^a^	0.08	<0.01
IgG, mg/mL	3.86 ^c^	4.72 ^b^	5.69 ^a^	0.19	<0.01
IgM, mg/mL	0.98 ^b^	1.11 ^b^	1.45 ^a^	0.05	<0.01
Lysozyme, μg/mL	12.0	11.7	11.7	0.48	0.94
immune factors					
IL-2, pg/mL	563 ^b^	726 ^a^	829 ^a^	26.9	<0.01
IL-6, pg/mL	130	128	132	4.17	0.91
IL-10, pg/mL	19.8 ^c^	32.6 ^b^	41.6 ^a^	2.07	<0.01
IFN-γ, μg/mL	0.79 ^c^	1.02 ^b^	1.29 ^a^	0.04	<0.01
C3, μg/mL	32.0 ^b^	38.8 ^b^	52.9 ^a^	1.96	<0.01
C4, μg/mL	101 ^c^	136 ^b^	174 ^a^	7.21	<0.01

SOD: superoxide dismutase; GSH-Px: glutathione peroxidase; T-AOC: total antioxidant capacity; MDA: malondialdehyde; IgA: immunoglobulin A; IgG: immunoglobulin G; IgM: immunoglobulin M; IL-2: interleukin-2; IL-6: interleukin-6; IL-10: interleukin-10; IFN-γ: interferon-γ; C3: complement C3; C4: complement C4. Different letters indicate significant differences between the groups (*p* < 0.05).

## Data Availability

The data presented in this study are available on request from the corresponding authors.
